# 2-{(1*E*)-1-[(3-{(*E*)-[1-(2-Hy­droxy-4-meth­oxy­phen­yl)ethyl­idene]amino}-2,2-di­methyl­prop­yl)imino]­eth­yl}-5-meth­oxy­phenol

**DOI:** 10.1107/S1600536811038815

**Published:** 2011-09-30

**Authors:** Akbar Ghaemi, Saeed Rayati, Ehsan Elahi, Seik Weng Ng, Edward R. T. Tiekink

**Affiliations:** a Department of Chemistry, Saveh Branch, Islamic Azad University, Saveh, Iran; b Department of Chemistry, K. N. Toosi University of Technology, PO Box, 16315-1618, Tehran, Iran; c Department of Chemistry, University of Malaya, 50603 Kuala Lumpur, Malaysia; d Chemistry Department, Faculty of Science, King Abdulaziz University, PO Box 80203 Jeddah, Saudi Arabia

## Abstract

Mol­ecules of the title compound, C_23_H_30_N_2_O_4_, are located on a crystallographic mirror plane. The mol­ecule has a curved shape with the dihedral angle formed between the two benzene rings being 55.26 (5)°. Intra­molecular O—H⋯N hydrogen bonds are noted. In the crystal, supra­molecular layers are formed in the *ac* plane owing to the presence of C—H⋯π inter­actions.

## Related literature

For our previous work on Schiff base complexes, see: Rayati *et al.* (2007 ▶, 2010 ▶).
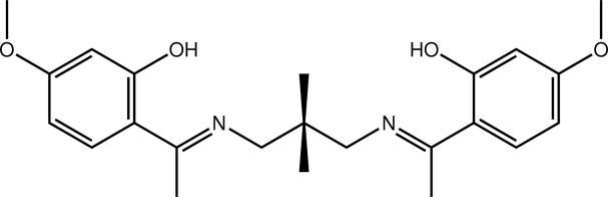


## Experimental

### 

#### Crystal data

C_23_H_30_N_2_O_4_
*M* *_r_* = 398.49
Orthorhombic, 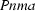

*a* = 10.0764 (7) Å
*b* = 36.069 (2) Å
*c* = 5.8322 (4) Å
*V* = 2119.7 (2) Å^3^
*Z* = 4
Mo *K*α radiation
μ = 0.09 mm^−1^
*T* = 294 K
0.30 × 0.25 × 0.20 mm

#### Data collection

Agilent SuperNova Dual diffractometer with Atlas detector
Absorption correction: multi-scan (*CrysAlis PRO*; Agilent, 2010 ▶) *T*_min_ = 0.793, *T*_max_ = 1.000
6816 measured reflections
2419 independent reflections
1952 reflections with *I* > 2σ(*I*)
*R*_int_ = 0.039

#### Refinement

*R*[*F* ^2^ > 2σ(*F* ^2^)] = 0.055
*wR*(*F* ^2^) = 0.155
*S* = 1.08
2419 reflections
141 parameters
1 restraint
H atoms treated by a mixture of independent and constrained refinement
Δρ_max_ = 0.23 e Å^−3^
Δρ_min_ = −0.18 e Å^−3^

### 

Data collection: *CrysAlis PRO* (Agilent, 2010 ▶); cell refinement: *CrysAlis PRO*; data reduction: *CrysAlis PRO*; program(s) used to solve structure: *SHELXS97* (Sheldrick, 2008 ▶); program(s) used to refine structure: *SHELXL97* (Sheldrick, 2008 ▶); molecular graphics: *ORTEP-3* (Farrugia, 1997 ▶) and *DIAMOND* (Brandenburg, 2006 ▶); software used to prepare material for publication: *publCIF* (Westrip, 2010 ▶).

## Supplementary Material

Crystal structure: contains datablock(s) global, I. DOI: 10.1107/S1600536811038815/bt5648sup1.cif

Structure factors: contains datablock(s) I. DOI: 10.1107/S1600536811038815/bt5648Isup2.hkl

Supplementary material file. DOI: 10.1107/S1600536811038815/bt5648Isup3.cml

Additional supplementary materials:  crystallographic information; 3D view; checkCIF report

## Figures and Tables

**Table 1:** Hydrogen-bond geometry (Å, °) *Cg*1 is the centroid of the C1–C6 ring.

*D*—H⋯*A*	*D*—H	H⋯*A*	*D*⋯*A*	*D*—H⋯*A*
O1—H1⋯N1	0.86 (1)	1.70 (2)	2.507 (2)	157 (4)
C7—H7c⋯*Cg*1^i^	0.96	2.75	3.547 (2)	141
C9—H9b⋯*Cg*1^ii^	0.96	2.66	3.456 (2)	140

Symmetry codes: (i) 
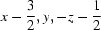
; (ii) 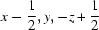.

**Fig. 1.**
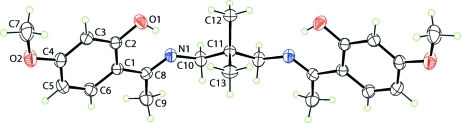
The molecular structure of (I) showing the atom-labelling scheme and displacement ellipsoids at the 50% probability level. The molecule has mirror symmetry and the unlabelled atoms are related by the symmetry operation x, 3/2 - y, z.

**Fig. 2.**
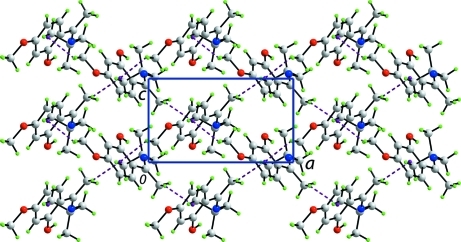
Supramolecular layer in the ac plane in (I) sustained by C—H···π interactions shown as purple dashed lines.

**Fig. 3.**
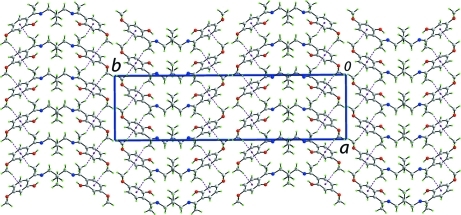
A view in projection down the c axis of the unit-cell contents of (I), highlighting the stacking of layers along the b axis. The C—H···π interactions are shown as purple dashed lines.

## supplementary crystallographic information

### Comment

The crystallographic investigation of the title compound, (I), was motivated by recent research in Schiff base complexes (Rayati *et al.*, 2007; Rayati *et al.*, 2010). The molecule of (I), Fig. 1, has crystallographically imposed mirror symmetry. The dihedral angle formed between the two benzene rings is 55.26 (5) ° indicating that, overall, the molecule has a curved shape. The presence of an intramolecular O—H···N hydrogen bond is noted, Table 1. The methoxy group is co-planar with the benzene ring to which it is attached as seen in the value of the C7—O2—C4—C3 torsion angle of -3.4 (3) °.

Molecules are assembled into layers in the *ac* plane through the agency of C—H···π interactions, Table 1 and Fig. 2, formed by methyl-H and the (C1—C6) benzene ring, indicating that the latter is bridging. Layers stack along the *b* axis, Fig. 3.

### Experimental

To a stirred ethanolic solution (30 ml) of 2,2-dimethylpropylenediamine (0.102 g, 1 mmol), 2-hydroxy-4-methoxyacetophenone (0.332 g, 2 mmol) was added. The bright-yellow solution was stirred and heated under reflux for 1 h. Crystals were obtained by evaporation of an ethanol solution of the product at room temperature. Yield: 85%; *M*.pt. 423 K. Selected FT—IR data (cm^-1^): 3427 ν(O—H), 2929–2965 ν(C—H), 1607 ν(C═N), 1446 ν(C═C), 1022 ν(C—O). ^1^H NMR (δ): 1.23 (s, 6H, C(CH_3_)_2_), 2.31 (s, 6H, OCH_3_C═ N), 3.47 (s, 4H, NCH_2_), 3.80 (s, 6H, OCH_3_), 6.24–7.37 (m, 6H, ArH), 12.35 (s, 2H, OH) p.p.m..

### Refinement

The C-bound H-atoms were placed in calculated positions (C—H 0.93 to 0.97 Å) and were included in the refinement in the riding model approximation, with *U*_iso_(H) set to 1.2 to 1.5*U*_equiv_(C).

### Crystal data

**Table:**

C_23_H_30_N_2_O_4_	*F*(000) = 856
*M**_r_* = 398.49	*D*_x_ = 1.249 Mg m^−^^3^
Orthorhombic, *P**n**m**a*	Mo *K*α radiation, λ = 0.71073 Å
Hall symbol: -P 2ac 2n	Cell parameters from 2488 reflections
*a* = 10.0764 (7) Å	θ = 2.3–27.5°
*b* = 36.069 (2) Å	µ = 0.09 mm^−^^1^
*c* = 5.8322 (4) Å	*T* = 294 K
*V* = 2119.7 (2) Å^3^	Prism, yellow
*Z* = 4	0.30 × 0.25 × 0.20 mm

### Data collection

**Table:**

Agilent SuperNova Dual diffractometer with Atlas detector	2419 independent reflections
Radiation source: SuperNova (Mo) X-ray Source	1952 reflections with *I* > 2σ(*I*)
Mirror	*R*_int_ = 0.039
Detector resolution: 10.4041 pixels mm^-1^	θ_max_ = 27.6°, θ_min_ = 3.4°
ω scan	*h* = −9→13
Absorption correction: multi-scan (*CrysAlis PRO*; Agilent, 2010)	*k* = −33→46
*T*_min_ = 0.793, *T*_max_ = 1.000	*l* = −5→7
6816 measured reflections	

### Refinement

**Table:**

Refinement on *F*^2^	Primary atom site location: structure-invariant direct methods
Least-squares matrix: full	Secondary atom site location: difference Fourier map
*R*[*F*^2^ > 2σ(*F*^2^)] = 0.055	Hydrogen site location: inferred from neighbouring sites
*wR*(*F*^2^) = 0.155	H atoms treated by a mixture of independent and constrained refinement
*S* = 1.08	*w* = 1/[σ^2^(*F*_o_^2^) + (0.0642*P*)^2^ + 0.9075*P*] where *P* = (*F*_o_^2^ + 2*F*_c_^2^)/3
2419 reflections	(Δ/σ)_max_ = 0.001
141 parameters	Δρ_max_ = 0.23 e Å^−^^3^
1 restraint	Δρ_min_ = −0.18 e Å^−^^3^

### Special details

**Table:**

Geometry. All s.u.'s (except the s.u. in the dihedral angle between two l.s. planes) are estimated using the full covariance matrix. The cell s.u.'s are taken into account individually in the estimation of s.u.'s in distances, angles and torsion angles; correlations between s.u.'s in cell parameters are only used when they are defined by crystal symmetry. An approximate (isotropic) treatment of cell s.u.'s is used for estimating s.u.'s involving l.s. planes.
Refinement. Refinement of *F*^2^ against ALL reflections. The weighted *R*-factor *wR* and goodness of fit *S* are based on *F*^2^, conventional *R*-factors *R* are based on *F*, with *F* set to zero for negative *F*^2^. The threshold expression of *F*^2^ > σ(*F*^2^) is used only for calculating *R*-factors(gt) *etc*. and is not relevant to the choice of reflections for refinement. *R*-factors based on *F*^2^ are statistically about twice as large as those based on *F*, and *R*- factors based on ALL data will be even larger.

### Fractional atomic coordinates and isotropic or equivalent isotropic displacement parameters (Å^2^)

**Table:**

	*x*	*y*	*z*	*U*_iso_*/*U*_eq_	Occ. (<1)
O1	0.30246 (15)	0.64954 (4)	0.1933 (2)	0.0531 (4)	
H1	0.354 (3)	0.6654 (8)	0.256 (6)	0.122 (13)*	
O2	0.13785 (15)	0.53184 (4)	0.4322 (3)	0.0574 (4)	
N1	0.46777 (15)	0.68176 (4)	0.4452 (3)	0.0419 (4)	
C1	0.39243 (16)	0.62163 (5)	0.5332 (3)	0.0352 (4)	
C2	0.30646 (17)	0.62129 (5)	0.3409 (3)	0.0372 (4)	
C3	0.22205 (18)	0.59119 (5)	0.3013 (3)	0.0409 (4)	
H3	0.1682	0.5908	0.1719	0.049*	
C4	0.21869 (18)	0.56202 (5)	0.4543 (3)	0.0422 (4)	
C5	0.3005 (2)	0.56209 (5)	0.6471 (3)	0.0477 (5)	
H5	0.2975	0.5425	0.7506	0.057*	
C6	0.38514 (19)	0.59118 (5)	0.6829 (3)	0.0436 (4)	
H6	0.4399	0.5908	0.8112	0.052*	
C7	0.0463 (2)	0.53147 (6)	0.2451 (5)	0.0660 (6)	
H7A	−0.0043	0.5089	0.2490	0.099*	
H7B	0.0942	0.5329	0.1031	0.099*	
H7C	−0.0125	0.5523	0.2576	0.099*	
C8	0.47861 (17)	0.65354 (5)	0.5787 (3)	0.0360 (4)	
C9	0.5749 (2)	0.65215 (6)	0.7746 (4)	0.0538 (5)	
H9A	0.6438	0.6701	0.7504	0.081*	
H9B	0.6134	0.6278	0.7836	0.081*	
H9C	0.5293	0.6576	0.9151	0.081*	
C10	0.54167 (19)	0.71600 (5)	0.4786 (4)	0.0460 (5)	
H10A	0.6219	0.7153	0.3869	0.055*	
H10B	0.5674	0.7181	0.6384	0.055*	
C11	0.4589 (2)	0.7500	0.4108 (4)	0.0342 (5)	
C12	0.4327 (3)	0.7500	0.1527 (4)	0.0441 (6)	
H12A	0.5157	0.7500	0.0719	0.066*	
H12B	0.3830	0.7717	0.1121	0.066*	0.50
H12C	0.3830	0.7283	0.1121	0.066*	0.50
C13	0.3269 (3)	0.7500	0.5407 (5)	0.0493 (7)	
H13A	0.3437	0.7500	0.7027	0.074*	
H13B	0.2770	0.7283	0.5005	0.074*	0.50
H13C	0.2770	0.7717	0.5005	0.074*	0.50

### Atomic displacement parameters (Å^2^)

**Table:**

	*U*^11^	*U*^22^	*U*^33^	*U*^12^	*U*^13^	*U*^23^
O1	0.0641 (9)	0.0448 (7)	0.0504 (8)	−0.0113 (7)	−0.0195 (7)	0.0126 (6)
O2	0.0598 (9)	0.0403 (7)	0.0721 (10)	−0.0103 (7)	−0.0096 (8)	0.0061 (7)
N1	0.0415 (8)	0.0358 (7)	0.0482 (8)	−0.0012 (6)	−0.0100 (7)	0.0021 (6)
C1	0.0334 (8)	0.0348 (8)	0.0375 (8)	0.0061 (7)	0.0004 (7)	−0.0005 (7)
C2	0.0387 (9)	0.0364 (8)	0.0365 (8)	0.0052 (7)	−0.0004 (7)	0.0000 (7)
C3	0.0405 (9)	0.0397 (9)	0.0425 (9)	0.0020 (8)	−0.0043 (8)	−0.0016 (7)
C4	0.0394 (9)	0.0352 (9)	0.0518 (10)	0.0020 (8)	0.0028 (8)	−0.0021 (8)
C5	0.0526 (11)	0.0398 (9)	0.0507 (10)	0.0033 (9)	−0.0001 (9)	0.0104 (8)
C6	0.0434 (10)	0.0439 (9)	0.0435 (9)	0.0057 (8)	−0.0071 (8)	0.0054 (8)
C7	0.0645 (14)	0.0506 (11)	0.0829 (16)	−0.0150 (11)	−0.0162 (13)	−0.0018 (12)
C8	0.0310 (8)	0.0375 (8)	0.0396 (8)	0.0071 (7)	−0.0012 (7)	−0.0025 (7)
C9	0.0536 (12)	0.0484 (10)	0.0594 (12)	0.0001 (9)	−0.0215 (10)	0.0064 (9)
C10	0.0389 (10)	0.0398 (9)	0.0594 (11)	−0.0008 (8)	−0.0164 (9)	0.0033 (8)
C11	0.0301 (11)	0.0377 (12)	0.0348 (11)	0.000	−0.0039 (9)	0.000
C12	0.0476 (14)	0.0482 (14)	0.0366 (12)	0.000	−0.0019 (11)	0.000
C13	0.0399 (14)	0.0658 (18)	0.0422 (14)	0.000	0.0010 (11)	0.000

### Geometric parameters (Å, °)

**Table:**

O1—C2	1.334 (2)	C7—H7C	0.9600
O1—H1	0.857 (10)	C8—C9	1.500 (2)
O2—C4	1.366 (2)	C9—H9A	0.9600
O2—C7	1.429 (3)	C9—H9B	0.9600
N1—C8	1.286 (2)	C9—H9C	0.9600
N1—C10	1.455 (2)	C10—C11	1.535 (2)
C1—C6	1.405 (2)	C10—H10A	0.9700
C1—C2	1.417 (2)	C10—H10B	0.9700
C1—C8	1.466 (2)	C11—C12	1.528 (3)
C2—C3	1.399 (2)	C11—C13	1.531 (3)
C3—C4	1.380 (3)	C11—C10^i^	1.535 (2)
C3—H3	0.9300	C12—H12A	0.9600
C4—C5	1.394 (3)	C12—H12B	0.9600
C5—C6	1.369 (3)	C12—H12C	0.9600
C5—H5	0.9300	C13—H13A	0.9600
C6—H6	0.9300	C13—H13B	0.9600
C7—H7A	0.9600	C13—H13C	0.9600
C7—H7B	0.9600		
			
C2—O1—H1	103 (3)	C8—C9—H9A	109.5
C4—O2—C7	117.70 (16)	C8—C9—H9B	109.5
C8—N1—C10	123.15 (15)	H9A—C9—H9B	109.5
C6—C1—C2	116.94 (16)	C8—C9—H9C	109.5
C6—C1—C8	122.15 (16)	H9A—C9—H9C	109.5
C2—C1—C8	120.81 (15)	H9B—C9—H9C	109.5
O1—C2—C3	117.90 (15)	N1—C10—C11	111.42 (14)
O1—C2—C1	121.50 (16)	N1—C10—H10A	109.3
C3—C2—C1	120.60 (16)	C11—C10—H10A	109.3
C4—C3—C2	120.01 (17)	N1—C10—H10B	109.3
C4—C3—H3	120.0	C11—C10—H10B	109.3
C2—C3—H3	120.0	H10A—C10—H10B	108.0
O2—C4—C3	124.15 (17)	C12—C11—C13	109.7 (2)
O2—C4—C5	115.47 (16)	C12—C11—C10	110.34 (14)
C3—C4—C5	120.38 (17)	C13—C11—C10	110.17 (14)
C6—C5—C4	119.53 (17)	C12—C11—C10^i^	110.34 (14)
C6—C5—H5	120.2	C13—C11—C10^i^	110.17 (14)
C4—C5—H5	120.2	C10—C11—C10^i^	106.04 (19)
C5—C6—C1	122.50 (17)	C11—C12—H12A	109.5
C5—C6—H6	118.8	C11—C12—H12B	109.5
C1—C6—H6	118.8	H12A—C12—H12B	109.5
O2—C7—H7A	109.5	C11—C12—H12C	109.5
O2—C7—H7B	109.5	H12A—C12—H12C	109.5
H7A—C7—H7B	109.5	H12B—C12—H12C	109.5
O2—C7—H7C	109.5	C11—C13—H13A	109.5
H7A—C7—H7C	109.5	C11—C13—H13B	109.5
H7B—C7—H7C	109.5	H13A—C13—H13B	109.5
N1—C8—C1	117.46 (15)	C11—C13—H13C	109.5
N1—C8—C9	122.87 (16)	H13A—C13—H13C	109.5
C1—C8—C9	119.67 (15)	H13B—C13—H13C	109.5
			
C6—C1—C2—O1	177.56 (17)	C2—C1—C6—C5	0.5 (3)
C8—C1—C2—O1	1.1 (3)	C8—C1—C6—C5	176.91 (17)
C6—C1—C2—C3	−2.0 (2)	C10—N1—C8—C1	176.40 (16)
C8—C1—C2—C3	−178.41 (16)	C10—N1—C8—C9	−3.9 (3)
O1—C2—C3—C4	−177.35 (17)	C6—C1—C8—N1	−172.62 (17)
C1—C2—C3—C4	2.2 (3)	C2—C1—C8—N1	3.6 (2)
C7—O2—C4—C3	−3.4 (3)	C6—C1—C8—C9	7.7 (3)
C7—O2—C4—C5	176.26 (19)	C2—C1—C8—C9	−176.04 (16)
C2—C3—C4—O2	178.73 (17)	C8—N1—C10—C11	−144.49 (18)
C2—C3—C4—C5	−0.9 (3)	N1—C10—C11—C12	−66.5 (2)
O2—C4—C5—C6	179.79 (17)	N1—C10—C11—C13	54.8 (2)
C3—C4—C5—C6	−0.5 (3)	N1—C10—C11—C10^i^	173.97 (11)
C4—C5—C6—C1	0.7 (3)		

Symmetry codes: (i) *x*, −*y*+3/2, *z*.

### Hydrogen-bond geometry (Å, °)

**Table:**

Cg1 is the centroid of the C1–C6 ring.

**Table:**

*D*—H···*A*	*D*—H	H···*A*	*D*···*A*	*D*—H···*A*
O1—H1···N1	0.86 (1)	1.70 (2)	2.507 (2)	157 (4)
C7—H7c···Cg1^ii^	0.96	2.75	3.547 (2)	141
C9—H9b···Cg1^iii^	0.96	2.66	3.456 (2)	140

Symmetry codes: (ii) *x*−3/2, *y*, −*z*−1/2; (iii) *x*−1/2, *y*, −*z*+1/2.
